# Distributed Information Compression for Target Tracking in Cluster-Based Wireless Sensor Networks

**DOI:** 10.3390/s16060937

**Published:** 2016-06-22

**Authors:** Shi-Kuan Liao, Kai-Jay Lai, Hsiao-Ping Tsai, Chih-Yu Wen

**Affiliations:** Department of Electrical Engineering, Graduate Institute of Communication Engineering, National Chung Hsing University, Taichung 402, Taiwan; cliffqoo@hotmail.com (S.-K.L.); justdoit0215@hotmail.com (K.-J.L.); hptsai@nchu.edu.tw (H.-P.T.)

**Keywords:** information compression, target tracking, wireless sensor network

## Abstract

Target tracking is a critical wireless sensor application, which involves signal and information processing technologies. In conventional target position estimation methods, an estimate is usually demonstrated by an average target position. In contrast, this work proposes a distributed information compression method to describe the measurement uncertainty of tracking problems in cluster-based wireless sensor networks. The leader-based information processing scheme is applied to perform target positioning and energy conservation. A two-level hierarchical network topology is adopted for energy-efficient target tracking with information compression. A Level 1 network architecture is a cluster-based network topology for managing network operations. A Level 2 network architecture is an event-based and leader-based topology, utilizing the concept of information compression to process the estimates of sensor nodes. The simulation results show that compared to conventional schemes, the proposed data processing scheme has a balanced system performance in terms of tracking accuracy, data size for transmission and energy consumption.

## 1. Introduction

With the limited capabilities in a sensor node, an important issue of target tracking is how to perform efficient information processing of the target [1]. Thus, in order to describe the target behaviors and incorporate statistical models for the task, the idea of hierarchically organizing the sensors may be used to provide network scalability and achieve energy conservation. To fulfill the task, one of the major concerns is that the original local sensed data may be pre-processed, since there may be a redundant part of it, and its data size is closely related to the energy consumption. Accordingly, the proposed scheme, the Cooperative Target tracking via Compressing Information (CTCI) algorithm, presents a new view of the result for the target tracking, which explores a new relationship between the processed data and estimation accuracy and further investigates the trade-off between the data distortion and energy consumption.

The goal of this work is to develop a distributed target tracking method by considering two perspectives: (1) load-balanced tracking and (2) improving estimation accuracy. The first perspective is to build up a load-balanced network architecture. The concept of leader-based network operation is used to automatically achieve sensor tasking, considering sensor residual energy level, target state and estimation accuracy. To avoid the ambiguity, a clusterhead and cluster members refer to the original network topology of the static cluster-based network. A leader and sub-cluster members refer to the sensor group for the tracking task. Local criteria may be used to select a cluster with the tracking responsibility. Afterwards, a sub-cluster of the corresponding cluster for the tracking task is formed by a leader, which can be a clusterhead or a cluster member in the original hierarchical network topology. Therefore, the Level 1 clustering utilizes a static clustering scheme to build up a basic network architecture, which may eliminate the overheads of dynamic network topology control. In the presence of the target, the Level 2 clustering utilizes a dynamic clustering scheme to adaptively form a sub-cluster for the tracking task. Thus, a hybrid static/dynamic clustering scheme is applied to achieve distributed target detection and tracking.

Since the sensed data have some uncertainty and redundancy inherently, the second perspective is to investigate the target characteristics, such that the presentation of certain supplementary information can be applied to improve estimation accuracy, as well as reduce the amount of transmitted data. Within the sub-cluster, the sensing nodes provide their local estimates through Bayesian particle filtering [2] and forward the pre-processed results to the leader, considering the Bayesian estimation to extract parameters from the Gaussian noise structure.

The major contributions and key features of this work are: (1) proposing a novel cooperative positioning approach to adaptively maintain the information of measurement uncertainties; (2) developing an information compression scheme for target tracking in a two-level hierarchical wireless sensor network, which allows the selected active sensors to utilize information compression for energy conservation (e.g., using a small number of samples to roughly reconstruct the estimation distribution); (3) one of the main advantages of the Bayesian framework is that the tasking sensor carries along a complete distribution of the estimates of the target position. Thus, the processed estimation distribution is inherently a measure of the accuracy of the positioning system. With proper settings to reduce the computational burden, a particle filter can be an accurate, practical and flexible location estimation technique. Our previous work [3] proposes a low-complexity indoor tracking system using Bayesian filtering with a few samples (20 samples) in a wireless sensor network. Moreover, [4] also presents a case study of applying particle filters to location estimation for ubiquitous computing and shows that it is practical to run particle filters on devices with limited capabilities.

The rest of the paper is organized as follows: Section 2 reviews tracking systems and compares and contrasts the related works with the proposed CTCI scheme. Section 3 depicts three data processing schemes. Section 4 estimates the data size of CTCI and presents an analysis to provide a lower bound of tracking accuracy. In Section 5, the performance evaluation is depicted with varying the system parameters. Finally, we summarize the conclusions in Section 6.

## 2. Literature Review

The authors in [5] propose a clustering-based routing protocol, Low-Energy Adaptive Clustering Hierarchy (LEACH), which distributes the energy consumption among the sensors in the network to enhance the lifetime of the network. The authors in [6] argue that their organization of clusters is triggered by the events of the monitor area, which means that the network operation is event-centric, and the clusters should be organized again according to the features of the event. The authors in [7] propose an energy-efficient distributed network scheme for phenomena detection, distinguishing between static sensors that belong to the original clusterhead and a dynamic set of sensors that belong to the phenomenon according to their readings, where the readings of the sensors are processed before transmission by using DFT for reducing the dimensionality of the data. The authors in [8,9,10,11] use the simple binary sensor for target tracking. The output of each sensor is only one bit, which means that the energy consumption of the binary sensor model is very low, but also this means there is limited information for the base station to localize the target.

The authors in [12] propose an approximation scheme, the Data Compressing-based Target Tracking Protocol (DCTTP), for target tracking with low overheads that may be practical in sensor networks. DCTTP [12] uses the distance measurements of sensor nodes to estimate the target location. The active sensors transmit their readings, including the estimated distances between them and the target and sensor ID to their clusterheads. The clusterheads gather the received data and then process the data in a centralized manner. The clusterhead processes the data by bitmap, which is a record indicating if the sensed data from a sensor can be approximated to the average value *μ* of the data gathered by the clusterhead within a given compressing bound (e.g., using the standard deviation *σ* to be the compressing bound). Therefore, if the value of a sensor reading is larger than the lower bound (μ-σ) and smaller than the upper bound (μ+σ), the data will be replaced by *μ*, and the bit set to “0”, which means the data are compressed to *μ*. If they are not, set the bit to “1”, and store the difference between the data and the average value for decompressing to the original data in the base station.

**Figure 1:**
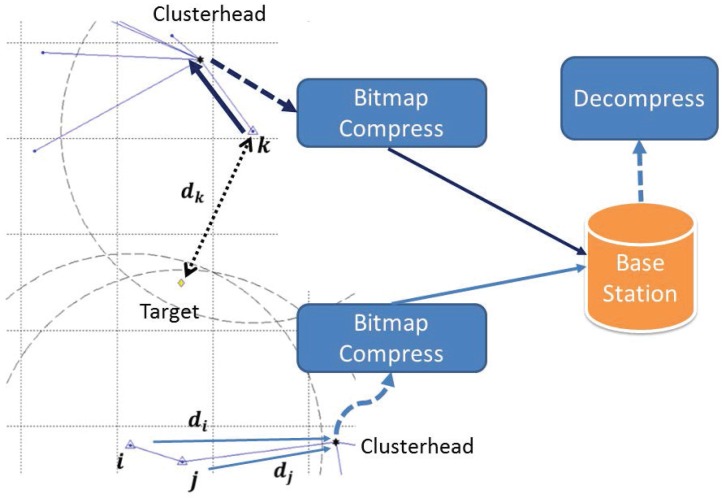
The result area of the Data Compressing-based Target Tracking Protocol (DCTTP).

After scanning all of the data, there are a binary sequence (*i.e.*, data compression) and a decimal sequence (*i.e.*, the difference between the data and *μ*) generated by the bitmap in the clusterhead. Then, the clusterhead transmits the sensor ID, average value and sequences of bitmaps to the base station. The base station decompresses the received data and uses them with the position of each active sensor to estimate the location of the target. The positions of active sensors and available information (e.g., distance measurements) form a possible area for the target (shown in Figure 1), which means the target positioning will be influenced by the position of active sensors. The rationale is that if the active sensor is closer to the target, its weighting for estimation will be larger. Then, the result of position estimation will move toward the active sensor. Moreover, the number and the relative positions of the active sensors with respect to the target also influence the result of the position estimation. That is, if the active sensors are dense to a side, the position estimate will be much closer to the active sensors, and it may make the estimate far away from the target.

In this work, for the information processing approach, we apply the concept of bitmap in [12] to compress the measurement information. The perspective of the binary sensor network inspires us to fully utilize the useful known information, which is not directly used by [13]. Accordingly, the CTCI method aims to balance the tracking performance in terms of estimation accuracy and energy consumption. In this paper, our previous work, the Two-level Clustering Approach via Timer (TCAT) [13] scheme, is used to build two-level clustering for the network architecture. Consequently, combining the Bayesian particle filter [14] and local neighboring information, the proposed CTCI scheme is able to efficiently complete the tracking task.

**Figure 2:**
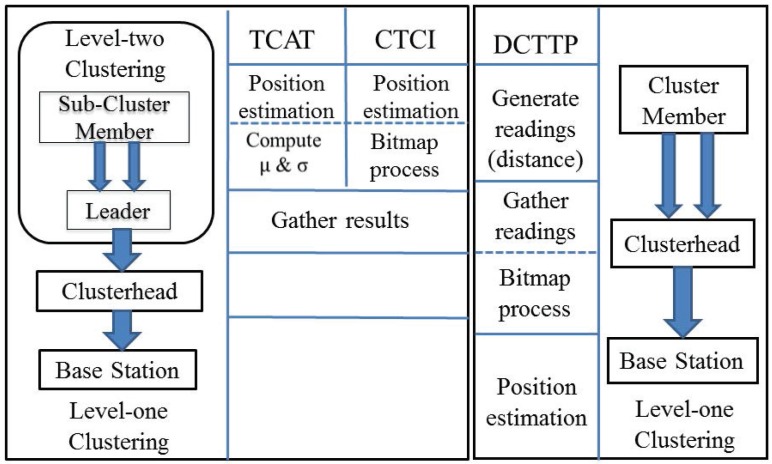
The information processing model of Two-level Clustering Approach via Timer (TCAT) (**left**), Cooperative Tracking via Compressing Information (CTCI) (**middle**) and DCTTP (**right**).

Here, we compare the information processing model of TCAT, CTCI and DCTTP for target tracking (shown in Figure 2). Observe that there is only a single hierarchy in DCTTP, but two hierarchies in TCAT. Thus, TCAT focuses on organizing the active sensors triggered by the target. Consequently, the system of TCAT is more stable and efficient than that of DCTTP, and TCAT can instantly acquire the result of target tracking at Level 2 clustering. Although there are many protocols proposed for target tracking, the network architecture and information processing problems are not considered simultaneously [7,8,9,10,11,15,16,17]. Hence, we tackle the tracking task and the data processing problem based on the network architecture in TCAT. Even though the energy consumption and the data size of CTCI are slightly higher than that of TCAT and DCTTP in some cases, the system of CTCI is able to adaptively provide the measurement distribution via information compression, not just describing the target position with a single estimate. Notice that the TCAT scheme can be considered as a special case of CTCI with a large compression bound, which compresses the samples to the mean. Moreover, the estimation accuracy and the integrality of the tracking result of CTCI are better than that of TCAT (improved by 20%) and are much better than that of DCTTP (improved by 100%). We investigate the trade-off between the tracking quality and the energy consumption in Section 5.

## 3. Data Processing Schemes

This section discusses the proposed data processing scheme, Cooperative Tracking via Compressing Information (CTCI), including the tracking method, information processing model, information flow and the meaning of the location estimation. First, we introduce the network architecture and TCAT, which is the basic model of the proposed CTCI data processing scheme. Secondly, we compare and contrast the DCTTP and TCAT schemes. Finally, a new data processing scheme, CTCI, is proposed to balance the distortion of the processed data and the energy consumption.

### 3.1. Problem Statement

**Figure 3:**
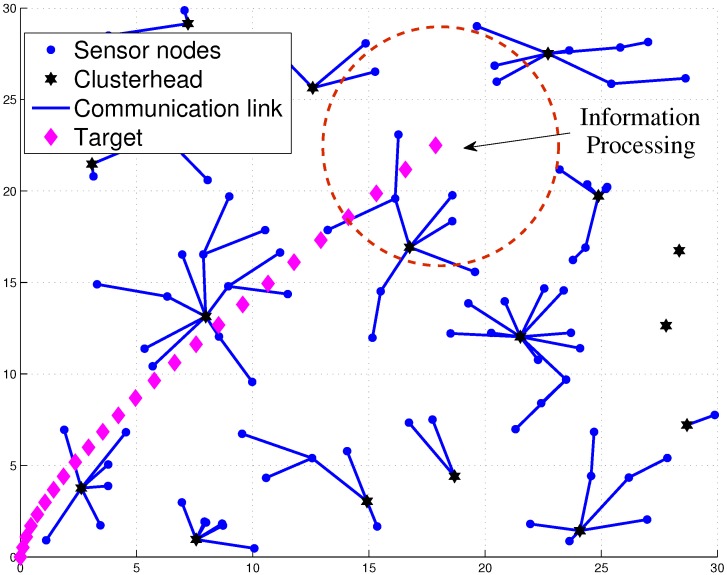
The cluster-based network topology and target movement with 25 time steps.

**Figure 4:**
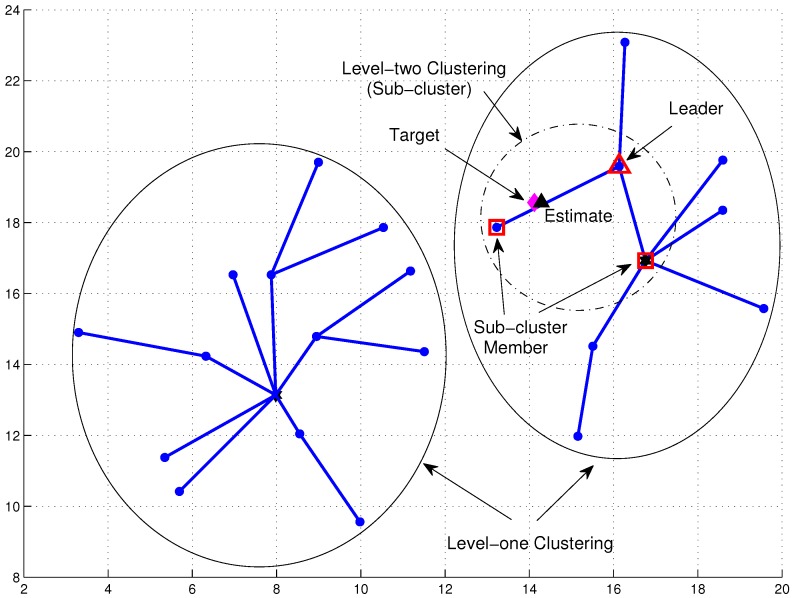
An example of Level 2 clustering for the tracking task.

Figure 3 shows an example of target movement with 25 time steps. In the presence of the target, to achieve load-balancing tracking, an important issue of target tracking is how to collect the desired data and to perform efficient information processing. Denote a sensor with tracking responsibility as an active sensor. Otherwise, a sensor is marked as an inactive sensor. Thus, referring to the Level 1 clustering in Figure 3, the Level 2 clustering utilizes a dynamic clustering scheme to adaptively form a local sub-cluster with active sensors for the tracking task in a distributed manner (Figure 4). Since there is a trade-off between estimation accuracy and energy consumption, this work aims to investigate the network performance on data size and energy conservation for target tracking from an information processing perspective. The main assumptions are: (1) all sensor nodes are assumed to be homogeneous with their fixed position information in a strongly-connected network; (2) the noise model is the zero-mean Gaussian noise measurement model; (3) Angle-of-Arrival (AOA) information or hybrid Time-of-Arrival (TOA)/AOA information is applied to perform target tracking; and (4) the target broadcasts a message for measurement purposes periodically. Note that these assumptions may be applied to locate objects (e.g., patients or animals) in healthcare or habitat monitoring scenarios.

**Figure 5:**
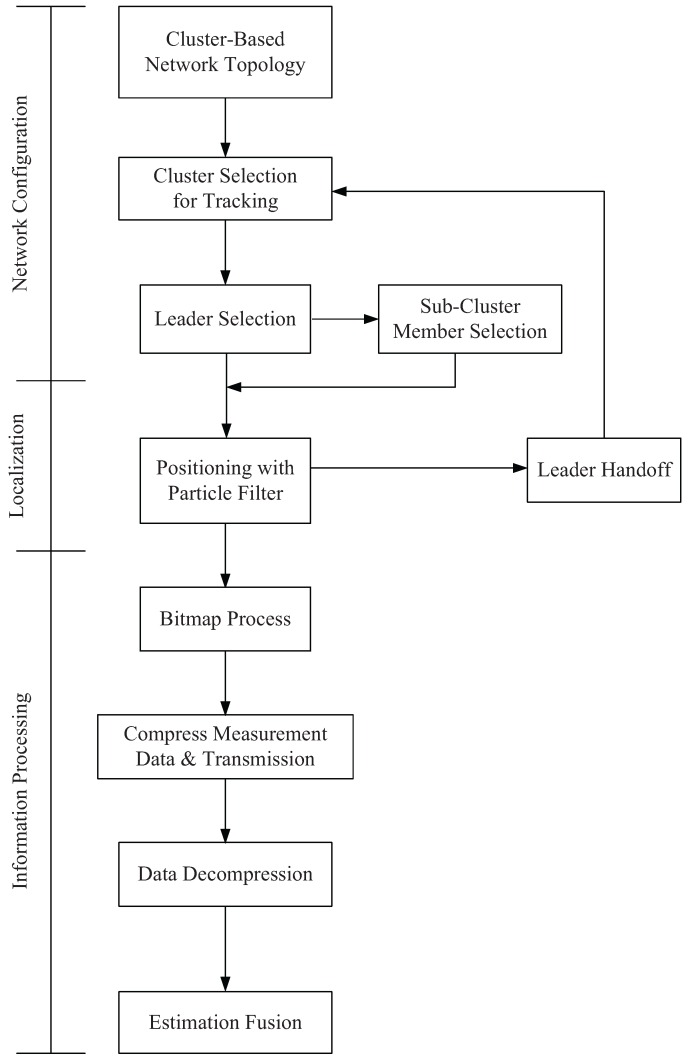
Illustration of the block diagram for the CTCI method.

Accordingly, as depicted in Figure 5, the CTCI scheme performs target tracking in three major phases: (1) network configuration; (2) target positioning; and (3) information processing. The following subsections describe each phase, respectively.

### 3.2. Network Configuration and TCAT

This section describes the fundamental system configuration of CTCI. To achieve distributed scheduling target detection and tracking, a two-level clustering (*i.e.*, a hybrid static/dynamic clustering scheme) is applied to deal with the control of network operations and to handle the tracking task, which is based on our previous works, the Clustering Algorithm via Waiting Timer (CAWT) scheme [18] and the TCAT scheme [13]. During the network initialization phase, sensors may apply the CAWT to establish a Level 1 network architecture (Figure 3). In order to handle the impact of the target movement, the TCAT scheme may be applied to form a sub-cluster with tracking responsibility and allow each sub-cluster member to locally locate the target in a distributed manner. Figure 4 shows an example of leader and sub-cluster member selection. Therefore, the TCAT scheme may be applied to select the sensors with tracking responsibility and build a Level 2 network architecture. Notice that the information flow is bi-directional (*i.e.*, it goes through the sub-cluster members to the leader and then to the clusterhead, and vice versa).

### 3.3. Target Positioning

**Figure 6:**
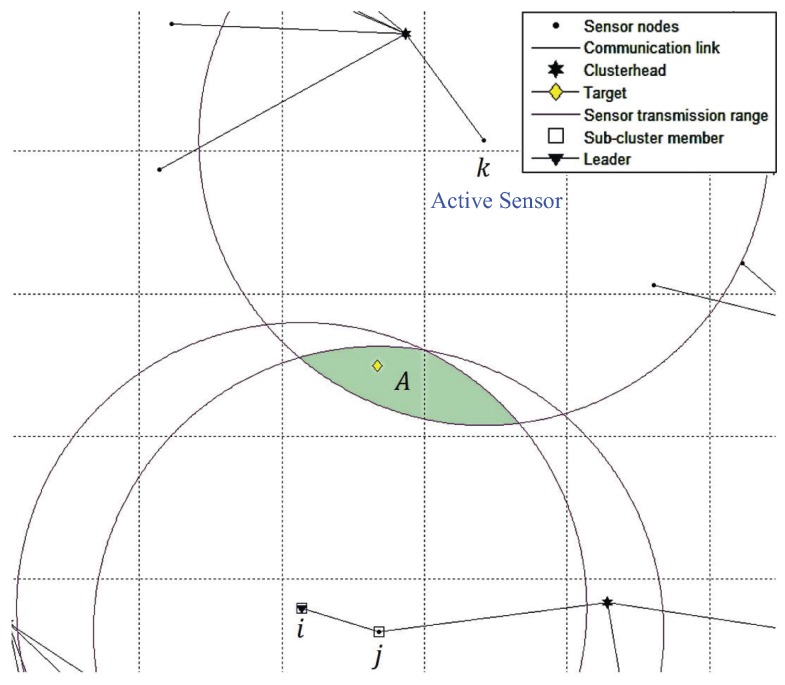
The bounding area A of CTCI.

**Figure 7:**
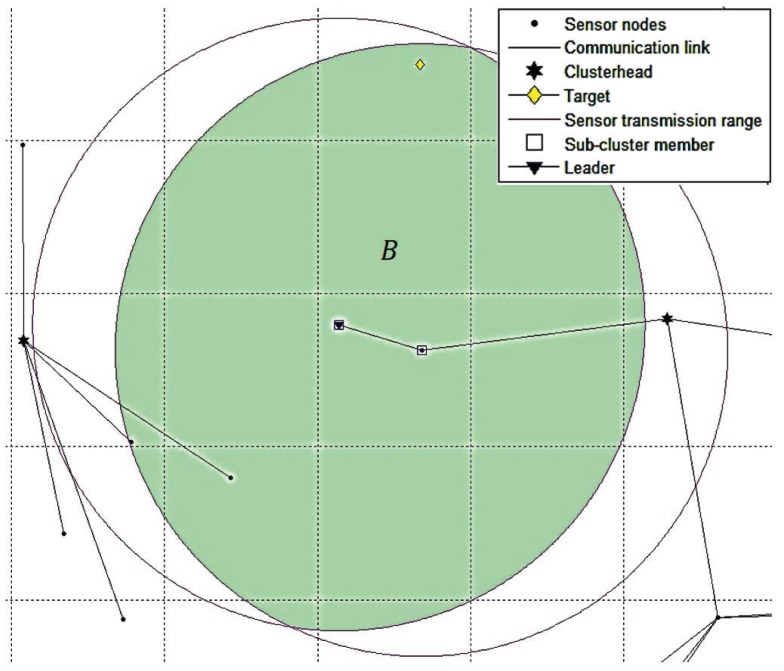
The bounding area B of TCAT.

**Figure 8:**
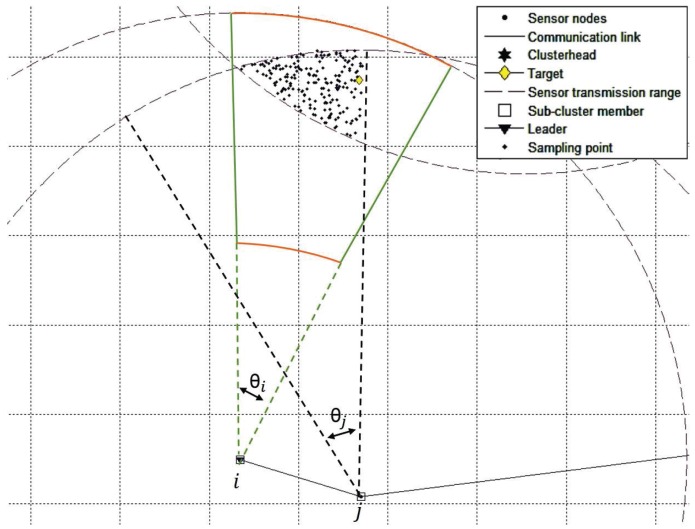
The intersection of sensor measurements and the area A, which leads to the distribution of the sampling points of CTCI.

The major difference between TCAT and CTCI is the bounding area formed by the different numbers of active sensors. In TCAT, the bounding area is determined by the active sensors in the same cluster. In CTCI, the bounding area is determined by not only the active sensors in a single cluster, but also the active sensors in other clusters. It is worth mentioning that including the active sensors in different clusters can effectively decrease the scope of the bounding area for sampling. The bigger the distance between the active sensor and the others, the smaller the intersection area of the transmission range of the active sensor will be, which suggests that we can provide a better sample area for particle filtering and even use fewer sampling points for the tracking task as the sample area decreases. Observe that in Figure 6, we can see that an active sensor node is added to form the bounding area A in CTCI, which is smaller than the bounding area B in TCAT (as shown in Figure 7). Accordingly, we may use a smaller number of samples (Figure 8) to achieve a comparable estimation accuracy with particle filtering.

On the basis of the refined sample area, the position estimation method is as follows:

1. Create a random sample $x_{k}\left(q\right)$, q=1,2,…,N from the refined sample area at time step k=0.
2. Each random sample is passed through the state Equation (1) to obtain samples at time step k+1.
  (1) $${\hat{x}}_{k+1}\left(q\right)=\Phi x_{k}\left(q\right)+\Gamma \lambda_{k}\left(q\right)$$
  where the system noise $\lambda_{k}\left(q\right)$ is a sample drawn from the Gaussian noise, Φ is characterized by the mobility model and Γ is assumed to be an identity matrix.
3. Upon receipt of the noisy measurement $z_{k+1}$, evaluate the likelihood of each prior sample and obtain the normalized weight of each sample. Therefore, after updating the weights of the likelihood function $p(z_{k+1}|{\hat{x}}_{k+1}\left(q\right))$ for each sample, the normalized weights yield:
  (2) $$\gamma_{k+1}\left(q\right)=\frac{p(z_{k+1}|{\hat{x}}_{k+1}\left(q\right))}{\sum_{j=1}^{N}p\left(z_{k+1}|{\hat{x}}_{k+1}\left(j\right)\right)}$$
4. Based on the normalized weights, it generates a new sample set $x_{k+1}\left(q\right)$ by performing sampling with replacement from the set ${\hat{x}}_{k+1}\left(q\right)$ (q=1,2,…,N), which describes the distribution of the position estimate.

Note that the noise model is the zero-mean Gaussian noise measurement model. A typical setting of the prior parameter values is described in Section 5.1.1. For obtaining the distribution of position estimate, each tasking sensor sends the mean and variance of the distribution to the tasking leader.

### 3.4. Information Processing and Data Management

**Figure 9:**
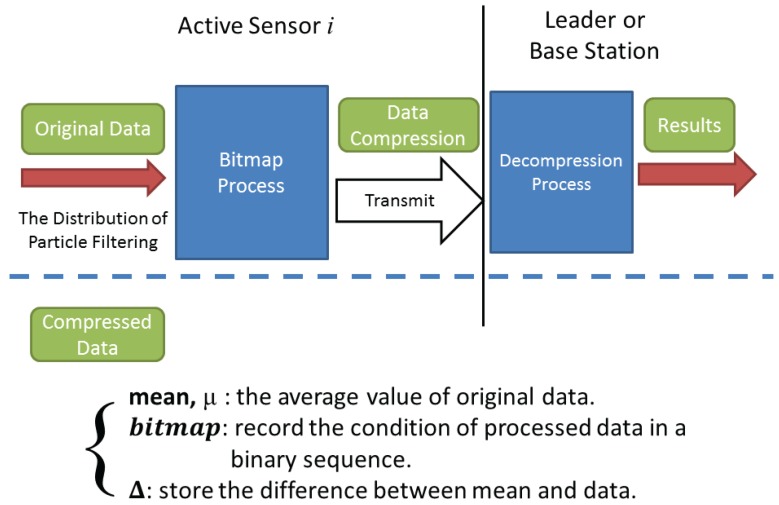
The conceptual processes of the CTCI scheme.

**Figure 10:**
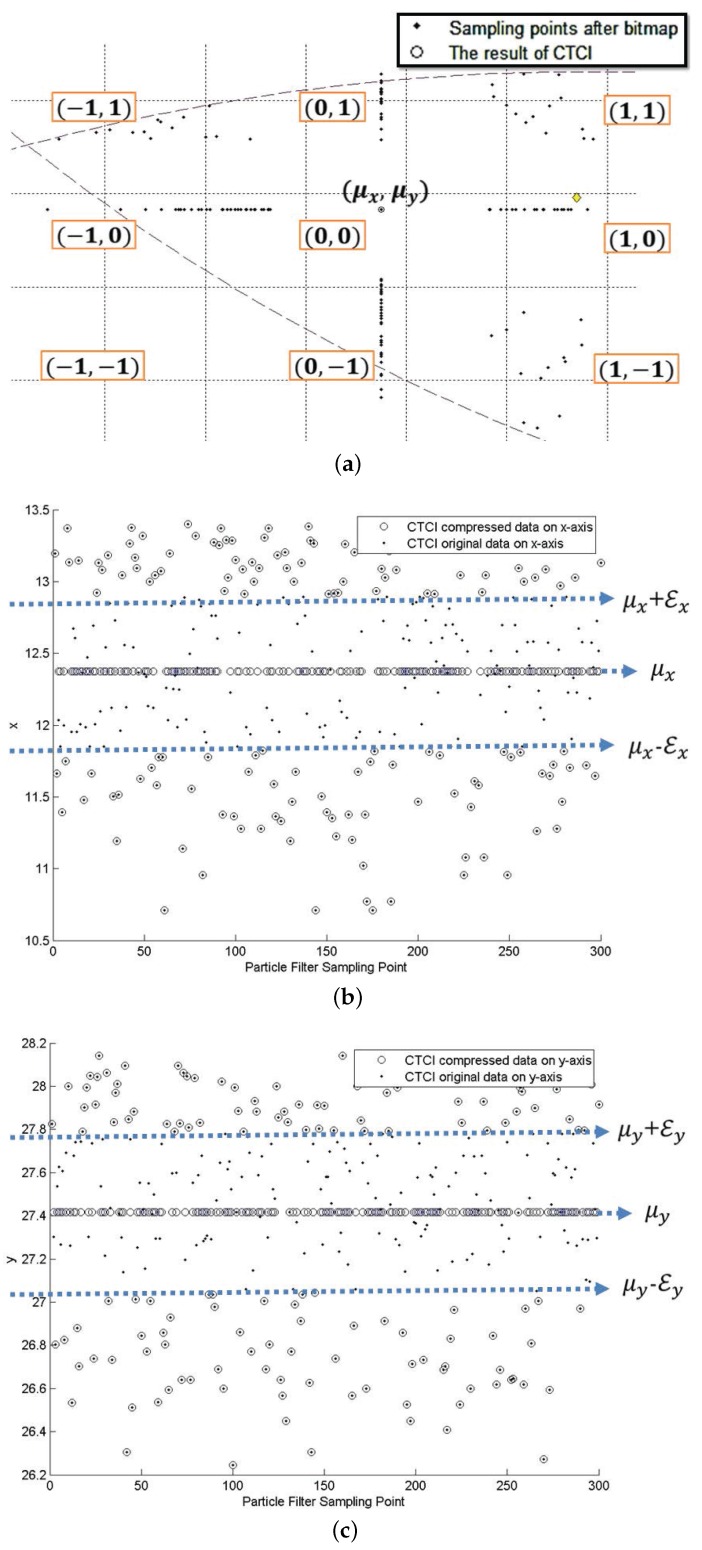
The sampling points after the bitmap process (**a**) and the impact of the compressing bound on distortion: *x*-axis (**b**) and *y*-axis (**c**).

**Figure 11:**
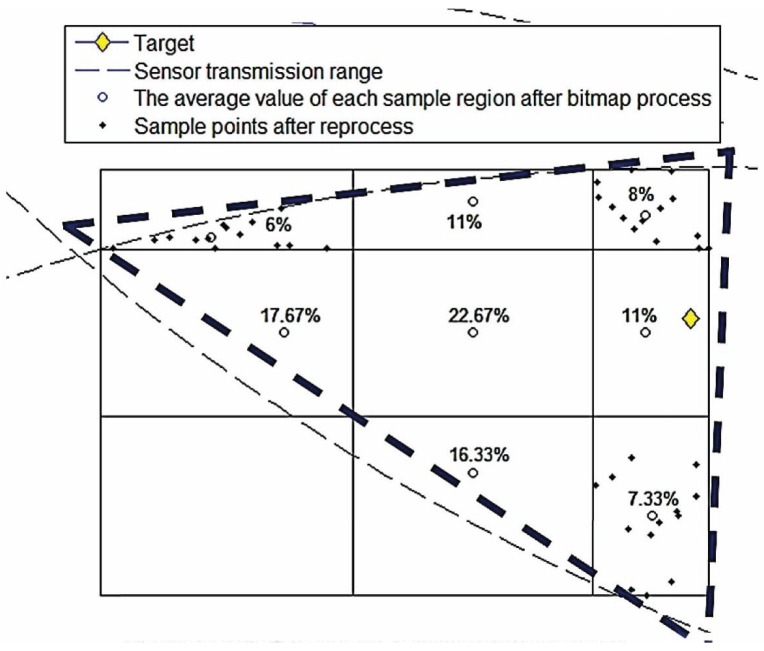
The external contour of the sample region and the distribution of the decompressed data.

Figure 9 depicts the conceptual procedures of information processing. The concepts and techniques (in the upper row) represent the processing mechanisms of active sensors and the leader, respectively. Upon obtaining the distribution of the target position by using a particle filter in an active sensor, data analysis is performed to explore the distribution properties, execute information compression and then transmit the processed data to a leader (or a base station). Afterwards, a leader (or a base station) may apply the received processed data (in the lower row) to generate a distribution, which roughly corresponds to the estimation distribution. The proposed algorithm uses the bitmap process and statistical properties to execute data processing, which consists of the following five steps:

1. *Preliminary settings*∼ Each tasking sensor performs target positioning with Bayesian filtering of *N* samples, which leads to a target position estimate at time step *k*, $P_{k}=\left(X_{k},Y_{k}\right)$, where $X_{k}=\left(x_{k}\left(1\right),x_{k}\left(2\right),\ldots ,x_{k}\left(N\right)\right)$, $Y_{k}=\left(y_{k}\left(1\right),y_{k}\left(2\right),\ldots ,y_{k}\left(N\right)\right)$.
2. *Configuring the bitmap of $P_{k}$*∼ Each tasking sensor computes the average ${\bar{\mu }}_{k}=\left({\bar{X}}_{k},{\bar{Y}}_{k}\right)$, $\sigma_{X_{k}}$, $\sigma_{Y_{k}}$, and generates the value of compressing bound, $\varepsilon_{X_{k}}=\sigma_{X_{k}}\cdot \eta$ and $\varepsilon_{Y_{k}}=\sigma_{Y_{k}}\cdot \eta$, where *η* is an adjustable factor to trade off the compression ratio and positioning accuracy.
3. *Compression of $\left(X_{k},Y_{k}\right)$*∼ As shown in Figure 10b,c, scan and compress $X_{k}$ and $Y_{k}$ (using $x_{k}\left(j\right)$ and $y_{k}\left(j\right)$ as examples). Hence, if ($|x_{k}\left(j\right)-{\bar{X}}_{k}|>\varepsilon_{X_{k}}$), then bit($x_{k}\left(j\right)$) = 1 and ${△}_{x_{k}\left(j\right)}=x_{k}\left(j\right)-{\bar{X}}_{k}$. Similarly, if ($|y_{k}\left(j\right)-{\bar{Y}}_{k}|>\varepsilon_{Y_{k}}$), then bit($y_{k}\left(j\right)$) = 1 and ${△}_{y_{k}\left(j\right)}=y_{k}\left(j\right)-{\bar{Y}}_{k}$. Afterwards, the tasking sensor (*i.e.*, a sub-cluster member) transmits the compressed data (${\bar{\mu }}_{k}$, △($X_{k}$), △($Y_{k}$), $\sigma_{X_{k}}$, $\sigma_{Y_{k}}$).
4. *Data decompression*∼ As shown in Figure 10a, decompress the received data by scanning ${\bar{\mu }}_{k}$, △($X_{k}$) and △($Y_{k}$). If bit($x_{k}\left(j\right)$) = 1, then $x_{k}\left(j\right)={\bar{X}}_{k}+{△}_{x_{k}\left(j\right)}$; otherwise, $x_{k}\left(j\right)={\bar{X}}_{k}$.
5. *Estimation fusion*∼ Figure 11 shows the external contour of the sample region and the distribution of the decompressed data. The leader node scans the information of each sample sub-region. Let $p_{m}$ be the target appearance probability of sample region *m*, which yields $p_{m}=N_{m}/N_{L}$ (m=1,2,…,9), where $N_{m}$ is the number of sample points in region *m* and $N_{L}$ is the total number of sample points. Let ${\bar{\mu }}_{k}\left(m\right)$ be the average estimate of sample region *m*. Accordingly, we obtain the location estimate $P_{L}=\sum_{m=1}^{9}p_{m}\cdot {\bar{\mu }}_{k}\left(m\right)$.

Therefore, the information processing of the CTCI scheme is completed.

Compared to DCTTP (a one-dimensional data processing method), the bitmap process in CTCI is a two-dimensional data processing method. Thus, the sampling points after the bitmap process will have several combinations the for *x* and *y* coordinates. From Figure 10a, we observe three basic combinations of the sampling points: (1) (0,0) means both the values of the *x* and *y* coordinates are approximated to the average value at the same time; (2) (1,0) or (0,1) means a value of the *x* or *y* coordinate is approximated to the average value, but the other one is not; (3) (1,1) means both values are not represented by the average value. The minus “−” means that the value of the *x* or *y* coordinate is smaller than the average value. Thus, the sample region is divided into nine parts by the bitmap process. In Figure 10b,c, the distributions of the original and compressed sampling points show the distortion of the *x* and *y* coordinates, respectively. The data within the compressing bound are represented to the average value, which causes the distortion. Thus, there is a trade-off between the energy consumption and the extent of distortion.

Therefore, each sub-cluster member transmits the compressed data, including (1) the average value and the standard deviation of the original sample points and (2) the differences between the sample points and the average value, to the leader node. Afterwards, the leader may transmit the position estimate to the base station through clusterheads and gateways in a multi-hop manner or directly disseminate the data from the clusterhead to the base station. In the leader node, the processed data are decompressed, and the external contour of the sample region is depicted by recovering the original sample points associated with (1,1), (−1,1), (1,−1), (−1,−1) representations as the results for data processing. The meaning of the result is shown in Figure 11, which means that the result of the target positioning is not only just a position estimation, but also a similar area of the original region for sampling. After obtaining the distribution of the position estimate, each tasking sensor sends the mean and variance of the distribution to the tasking leader.

## 4. Performance Analysis

This section performs an analysis of the data size and describes a conceptual-stochastic distribution of the position estimation.

### 4.1. Analysis of the Data Size

In order to assess the energy consumption during the data processing procedures, the data size is estimated, assuming that the sensor nodes transmit data in a binary system. Instead of using the floating point representation like IEEE standards, which reserve bits for exponents to represent a wider range of numbers, we use fixed point representation, which may cost less bits than floating point representation. This is because we only transmit the compressed data, *i.e.*, $\Delta_{x_{i}}$ and $\Delta_{y_{i}}$, which are the differences of $x_{i_{j}}$ and $\bar{X_{i}}$ and $y_{i_{j}}$ and $\bar{Y_{i}}$, respectively. Since the values of the differences are usually small, our algorithm can dynamically decide the number of bits for the integer part and reserve more bits for the fractional part, depending on the value and the required precision. Specifically, before transmitting the data stream, active sensor nodes scan the data stream first to find the maximum of it, which decides the largest bit number of the integer part and the position of the decimal point.

For instance, in Table 1, the first bit represents the sign of the value; the second bit to fourth bit specify the position of the decimal point; the fifth to seventh bit represent the integer part of the value; the eight to 16th bit represent the fractional part of the value; the code line is the bit stream to represent -5.678. Thus, if the required precision is to $2^{-5}$, we only use 12 bits to represent a value in that case. Observe that, in the example of Table 1, using IEEE standard with nine-bit mantissa to represent the difference leads to an error between the original value and the value after processing lower than $2^{-7}$. In contrast, our approach can achieve better precision with an error lower than $2^{-9}$.

### 4.2. Analysis of Positioning Accuracy

As the Cramer–Rao lower bound (CRLB) tests the performance of the proposed measurement mechanism, this subsection describes the achievable tracking accuracy of joint TOA/AOA positioning. The measurements at the reference sensor can be modeled as:

(3) $$\begin{array}{ccc} \hat{\tau } & = & \tau +\delta_{\tau } \end{array}$$

(4) $$\begin{array}{ccc} \hat{\phi } & = & \phi +\delta_{\phi } \end{array}$$

where *τ* is the true propagation time and *φ* is the true angle information. Note that $\delta_{\tau }$ and $\delta_{\phi }$ are uncorrelated Gaussian noises with the distributions $\delta_{\tau }∼N\left(0,\sigma_{\tau }^{2}\right)$ and $\delta_{\phi }∼N\left(0,\sigma_{\phi }^{2}\right)$. Assuming that the direct path exists between the sensor and the target, the estimated target position is given by:

(5) $$\begin{array}{ccc} \hat{x} & = & x_{s}+v\hat{\tau }\cos\left(\hat{\phi }\right)=x_{s}+\hat{r}\cos\left(\hat{\phi }\right) \end{array}$$

(6) $$\begin{array}{ccc} \hat{y} & = & y_{s}+v\hat{\tau }\sin\left(\hat{\phi }\right)=y_{s}+\hat{r}\sin\left(\hat{\phi }\right) \end{array}$$

where $\hat{r}$ is the distance measurement (*i.e.*, $\hat{r}=v\hat{\tau }=r+v\delta_{\tau }$), $\left(x_{s},y_{s}\right)$ is the true position of the sensor and *v* is the speed of the signal. Assuming $\delta_{\tau }$ and $\delta_{\phi }$ are sufficiently small, the variance of the position estimation $\hat{p}$ is approximated by:

(7) $$\sigma_{p}^{2}\approx v^{2}\sigma_{\tau }^{2}+d^{2}\sigma_{\phi }^{2}=\sigma_{r}^{2}+d^{2}\sigma_{\phi }^{2}$$

Given the above assumptions [19], the CRLB with a single sensor is derived as follows.

**Figure 12:**
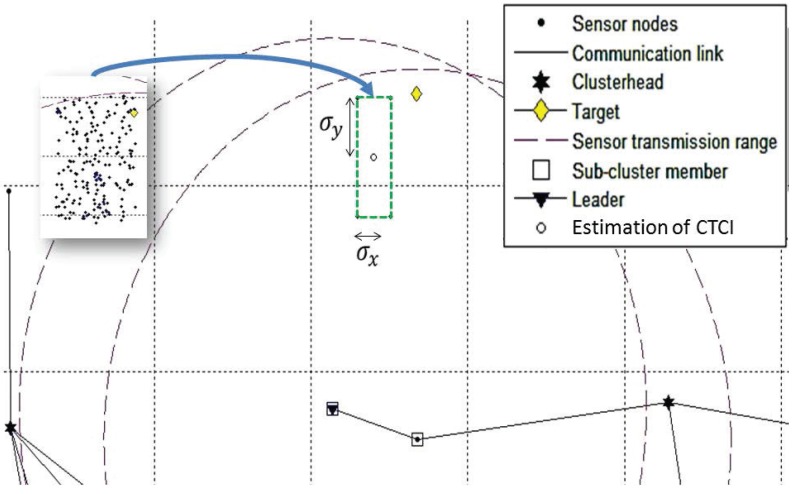
The stochastic distribution of position estimation.

Assuming that a line-of-sight path exists between a sensor and the target, the joint density function of the distance measurement $\hat{r}$ and the angle measurement $\hat{\phi }$ yields:

(8) $$\begin{array}{c} f\left(g;x,y\right)=\frac{1}{\sqrt{2\pi \sigma_{r}^{2}}}\cdot \exp\left[-\frac{1}{2\sigma_{r}^{2}}{\left(\hat{r}-d\right)}^{2}\right] \end{array}\;\cdot \frac{1}{\sqrt{2\pi \sigma_{\phi }^{2}}}\cdot \exp\left[-\frac{1}{2\sigma_{\phi }^{2}}{\left(\hat{\phi }-\arctan\left(\frac{y-y_{s}}{x-x_{s}}\right)\right)}^{2}\right]$$

where $g=[\hat{r},\hat{\phi }]$, $\left(x_{s},y_{s}\right)$ is the true sensor position and *d* is the true distance between pairwise sensors. Thus, the Fisher information matrix is given by:

(9) $$I\left(x^{\left(t\right)}\right)=\left[\begin{array}{cc} \frac{\cos^{2}\left(\phi \right)}{\sigma_{r}^{2}}+\frac{\sin^{2}\left(\phi \right)}{d^{2}\sigma_{\phi }^{2}} & \frac{\sin(2\phi )}{2}\left[\frac{1}{\sigma_{r}^{2}}-\frac{1}{d^{2}\sigma_{\phi }^{2}}\right]\\ \frac{\sin(2\phi )}{2}\left[\frac{1}{\sigma_{r}^{2}}-\frac{1}{d^{2}\sigma_{\phi }^{2}}\right] & \frac{\sin^{2}\left(\phi \right)}{\sigma_{r}^{2}}+\frac{\cos^{2}\left(\phi \right)}{d^{2}\sigma_{\phi }^{2}} \end{array}\right]$$

which leads to the CRLB for the best possible estimation for target tracking. Figure 12 shows a typical run of the conceptual-stochastic distribution of the position estimation. The readers may refer to [19] for details.

## 5. Simulation

This section describes the simulation settings and discusses the influence of simulation parameters on the tracking task. The simulation is conducted with various experimental settings (e.g., different network density, the communication range and network topology). The MATLAB© (MathWorks, Natick, MA, USA) simulation tool is applied to perform the comparison among our proposed approach CTCI, DCTTP [12] and TCAT [13]. The following subsections respectively describe the parameters, mobility model and energy consumption model used.

### 5.1. The Parameters

**Figure 13:**
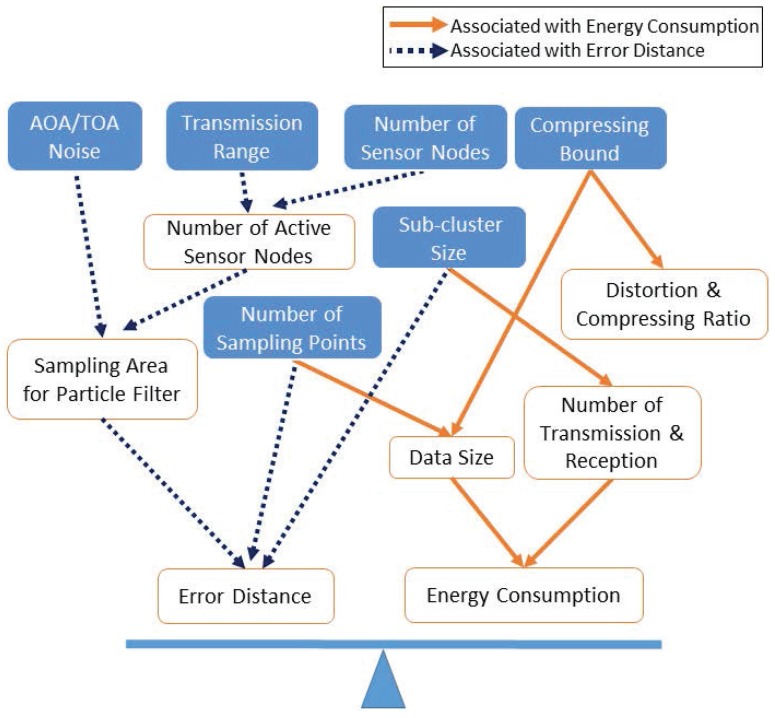
The impacts of system parameters on the tracking performance from two perspectives: (1) distance estimation and (2) energy conservation.

As illustrated in Figure 13, system parameters fall basically into two categories: Category I is associated with distance estimation and tracking: (a)AOA/TOA measurement noise; (b) transmission range; (c) number of sensor nodes; (d) compressing bound; (e) number of sub-cluster size; and (f) number of sampling points; Category II is associated with energy conservation: (1) the number of active sensor nodes; (2) the sampling area for the particle filter; (3) the distance estimation error; (4) distortion and compressing ratio; (5) data size; (6) the number of transmissions and receptions; and (7) the energy consumption. Figure 13 shows the impacts of system parameters on the tracking performance, such as distance estimation and energy conservation. Table 2 depicts the values of the simulation parameters for the target position estimate.

#### 5.1.1. Mobility Model

Suppose that the target movement within the x-y sensing field is based on the standard second-order model [14]:

(10) $$X_{k}=\Phi X_{k-1}+\Gamma w_{k}$$

over a four-dimensional state space, where $X_{k}={\left(x,\dot{x},y,\dot{y}\right)}_{k}^{T}$, $w_{k}={\left(w_{x},w_{y}\right)}_{k}^{T}$. Note that *x* and *y* denote the Cartesian coordinate of the target, and the uncertainties are described by uncorrelated Gaussian diffusion terms,

$$\Phi =\left(\begin{array}{cccc} 1 & 1 & 0 & 0\\ 0 & 1 & 0 & 0\\ 0 & 0 & 1 & 1\\ 0 & 0 & 0 & 1 \end{array}\right),\mathrm{and}\;\Gamma =\left(\begin{array}{cc} 0.5 & 0\\ 1 & 0\\ 0 & 0.5\\ 0 & 1 \end{array}\right)$$

Assuming that the measurement is contaminated by noise, the noisy measurement is represented by:

(11) $$z_{k}=\tan^{-1}\left(y_{k}/x_{k}\right)+v_{k}$$

where the measurement noise, $v_{k}$, is a zero mean Gaussian white noise process with a finite variance $\sigma_{\theta }^{2}$. Moreover, the system initial state is given by a Gaussian distribution with known mean ${\bar{x}}_{1}$ and covariance:

$$M_{1}=\left(\begin{array}{cccc} \sigma_{1}^{2} & 0 & 0 & 0\\ 0 & \sigma_{2}^{2} & 0 & 0\\ 0 & 0 & \sigma_{3}^{2} & 0\\ 0 & 0 & 0 & \sigma_{4}^{2} \end{array}\right)$$

The target trajectory monitoring and measurements are generated based on Equations (10) and (11). The values of the parameters are the following: the covariance of the system noise, $Q=qI_{2}$, where $I_{2}$ is the 2 × 2 identity matrix, $\sqrt{q}=0.001$. The target initial state is $x_{1}={\left(0.0,0.6,0.0,2.5\right)}^{T}$. Referring to the settings in [14], the prior parameter values are set to ${\bar{x}}_{1}={\left(0.0,0.0,0.4,0.05\right)}^{T}$, $\sigma_{1}=0.5$, $\sigma_{2}=0.001$, $\sigma_{3}=0.05$ and $\sigma_{4}=0.01$.

#### 5.1.2. Energy Consumption Model

In order to further assess the network performance, we explore the communication cost for the tracking task. Since the computational cost in a wireless sensor network is usually neglected compared to the communication cost, we focus on the analysis of power consumption for communication. Currently, there have been a number of studies on measuring the energy dissipation per transmitted bit [5,20]. Here, we adopt the energy model presented in [5] to describe the energy dissipation. Thus, the energy dissipation of a transmitter $E_{Tx}$ and a receiver $E_{Rx}$ can be modeled as follows [5]:

(12) $$\begin{array}{ccc} E_{Tx} & = & \left\{\begin{array}{cc} tE_{elec}+t\varepsilon_{fs}d^{2}, & d<d_{o}\\ tE_{elec}+t\varepsilon_{mp}d^{4}, & d\geq d_{o} \end{array}\right. \end{array}$$

(13) $$\begin{array}{ccc} E_{Rx} & = & tE_{elec} \end{array}$$

where *t* is the data packet size, $E_{elec}$ denotes the energy consumption of the electronic circuitry, $\varepsilon_{fs}$ and $\varepsilon_{mp}$ depend on distance *d* between the transmitter and the receiver for maintaining an acceptable bit-error rate and $d_{o}$ is a transmission threshold. The values of the simulation parameters are set as [5]: $E_{elec}$ = 50 nJ/bit, $\varepsilon_{fs}=10\mathrm{pJ}/\mathrm{bit}/m^{2}$, $\varepsilon_{mp}=0.0013\mathrm{pJ}/\mathrm{bit}/m^{4}$, $d_{o}$ is the transmission range *R*. Observe that the key to the energy consumption is the transmission distance and the data size. In our case, given a fixed transmission distance, how to reduce the data size for transmission becomes an important issue.

### 5.2. Number of Sensor Nodes

**Figure 14:**
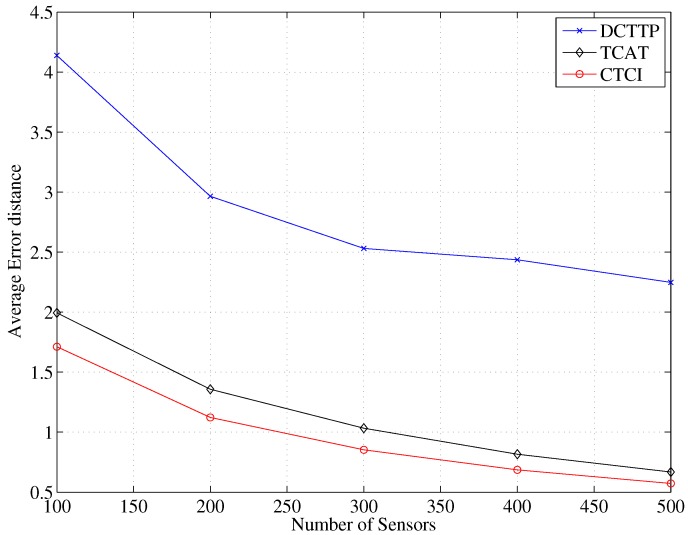
Impact of the number of sensor nodes on average error distance.

Figure 14 depicts the error distance of each scheme, which decreases as the number of the sensor nodes increases, which implies that if there are more active sensor nodes participating in target tracking, a larger amount of sensed data will be generated. For DCTTP with a larger number of active sensor nodes surrounding the target, the result of the target tracking will be improved. Because DCTTP uses centroid tracking, the base station receives the distance estimation of each active sensor node, (*i.e.*, the distance between each active sensor node and the target) and then uses the position of the active sensor nodes for target tracking. If the active sensor nodes are closer to the target and uniformly distributed, the performance of target tracking can be improved. On the other hand, if active sensor nodes are distributed in a certain direction, the result of the target tracking will move toward where the distributed active sensor nodes are, not to the target position. Therefore, the error distance of DCTTP may decrease with an increasing sensor density in the network.

In TCAT, with a higher sensor density (*i.e.*, a larger number of active sensor nodes in the cluster), the bounding area for sampling will be smaller, which leads to a lower error distance of TCAT. Comparing the method of target tracking of TCAT to that of DCTTP, TCAT uses multiple sensors with TOA/AOA information and a particle filter, and DCTTP just uses the distance estimation and the position of active sensor nodes. As shown in Figure 14, the error distance of TCAT is much lower than the error distance of DCTTP. In the proposed CTCI, with a larger sensor density, the bounding area for sampling points will be refined to a smaller region compared to TCAT. This is because the location information of active sensors can be used to further refine the sampling area. Therefore, the error distance of CTCI is lower than the error distance of TCAT.

**Figure 15:**
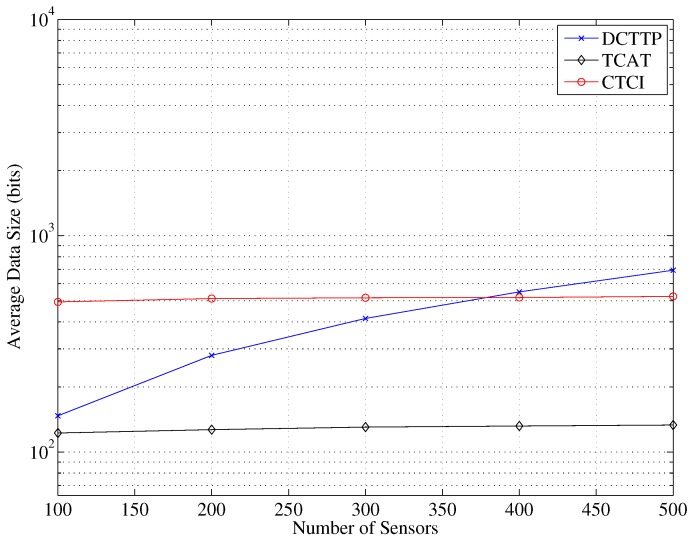
Impact of the number of sensor nodes on the average data size.

**Figure 16:**
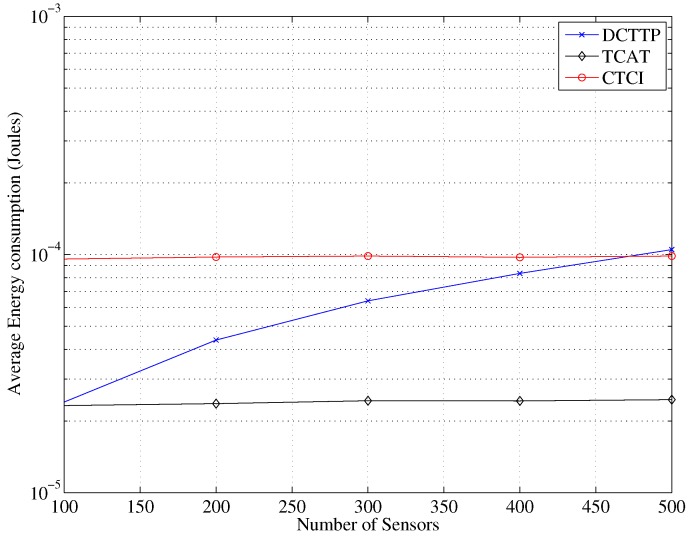
Impact of the number of sensor nodes on the average energy consumption.

Figure 15 depicts that the data size of DCTTP rapidly rises as the number of sensor nodes increases. Observe that when the number of sensor nodes is about 230, the data size of DCTTP is larger than that of TCAT. This is because the DCTTP does not have a constraint on the number of active sensors that participate in the tracking task. In contrast, the data size of TCAT and CTCI is nearly constant because of the pre-defined sub-cluster size. As shown in Figure 16, the distribution of the average energy consumption is similar to the performance of the average data size. Observe that with a high network density (e.g., the number of sensor nodes = 500), the energy consumption of CTCI is larger than that of TCAT and lower than that of DCTTP.

### 5.3. Transmission Range

**Figure 17:**
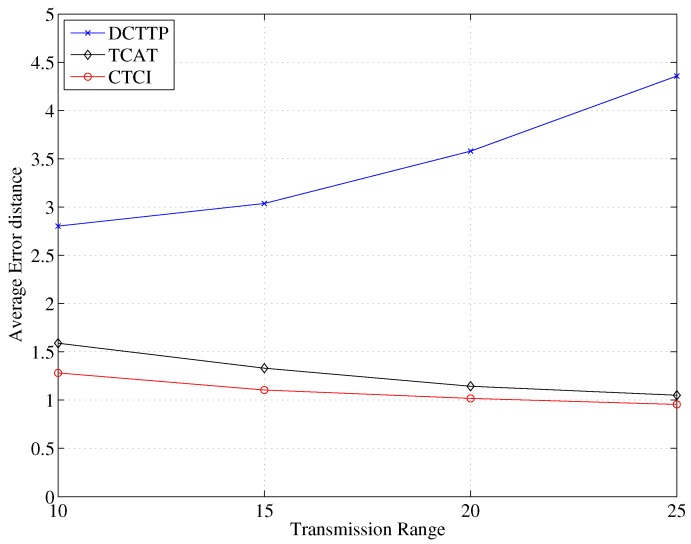
Impact of transmission range on the average error distance.

Figure 17 depicts that the error distance of TCAT and CTCI decreases, but the error distance of DCTTP increases rapidly as the transmission range increases. Because the location method of DCTTP is centroid positioning, the result of the positioning will be influenced by the position of the active sensor nodes. When the transmission range increases, there are more active sensor nodes that are far from the target. The result of the positioning is far away from the target; therefore, the error distance of DCTTP is larger.

**Figure 18:**
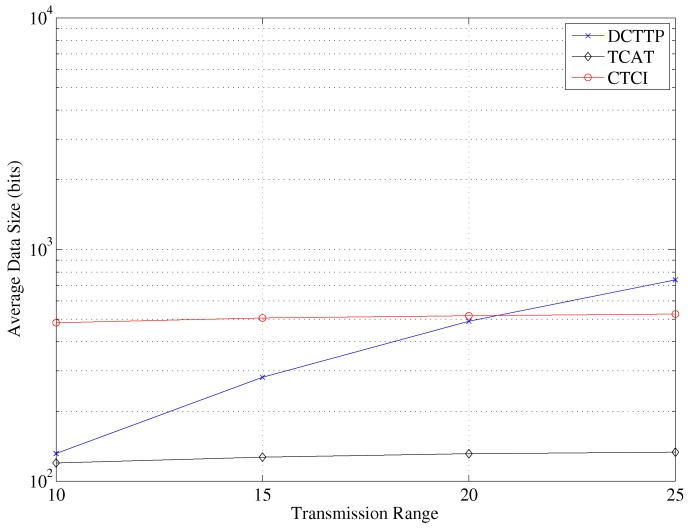
The impact of the transmission range on the average data size.

**Figure 19:**
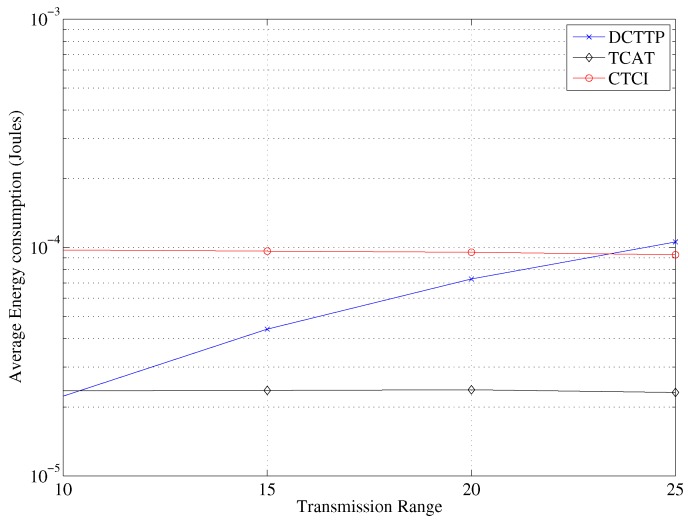
Impact of the transmission range on the average energy consumption.

In TCAT, the cluster size increases as the transmission range increases. For position estimation of TCAT, a larger cluster size may results in a larger number of active sensor nodes in the cluster to which the leader belongs. Therefore, the error distance decreases as the bounding area for sampling is smaller. Similarly, there are more active sensor nodes participating in refining the bounding area in CTCI than in TCAT, so the error distance of CTCI is smaller. Figure 18 depicts that the data size and the energy consumption increase rapidly as the transmission range increases. Figure 19 shows that when the transmission range is larger than 25, the energy consumption of CTCI is larger than that of TCAT and smaller than that of DCTTP.

### 5.4. Number of Sample Points

**Figure 20:**
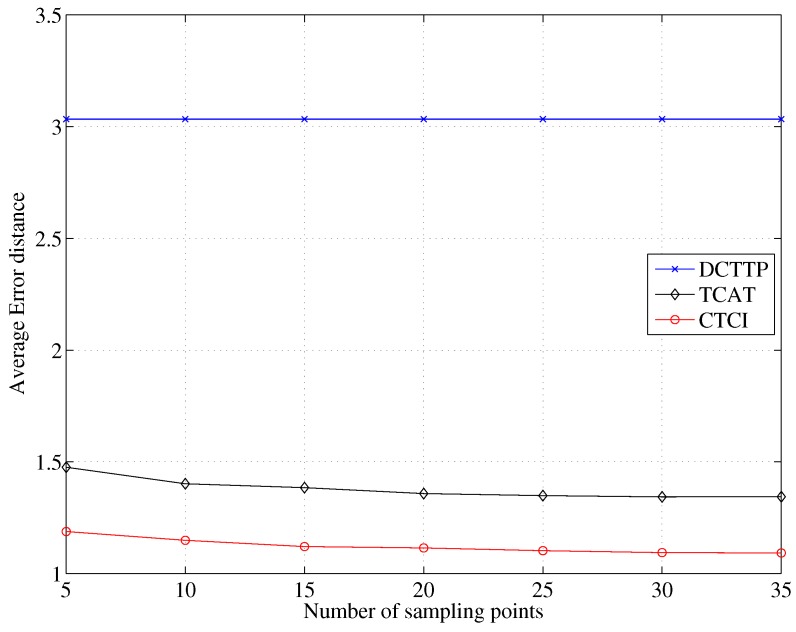
Impact of the sampling points on the average error distance.

**Figure 21:**
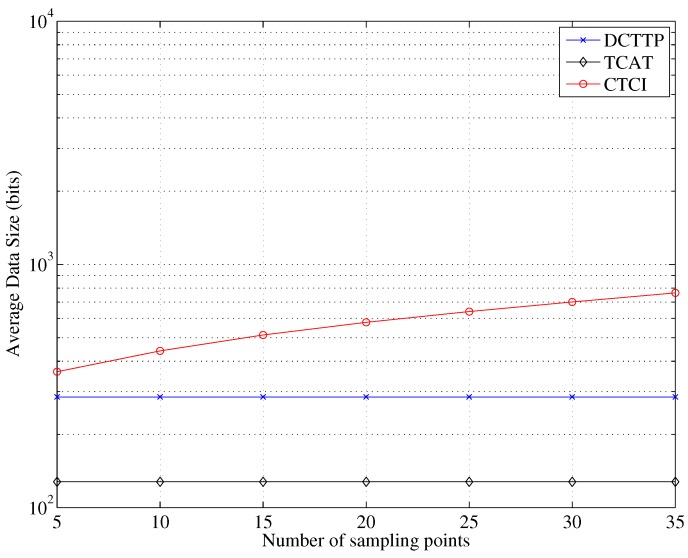
Impact of the sampling points on the average data size.

**Figure 22:**
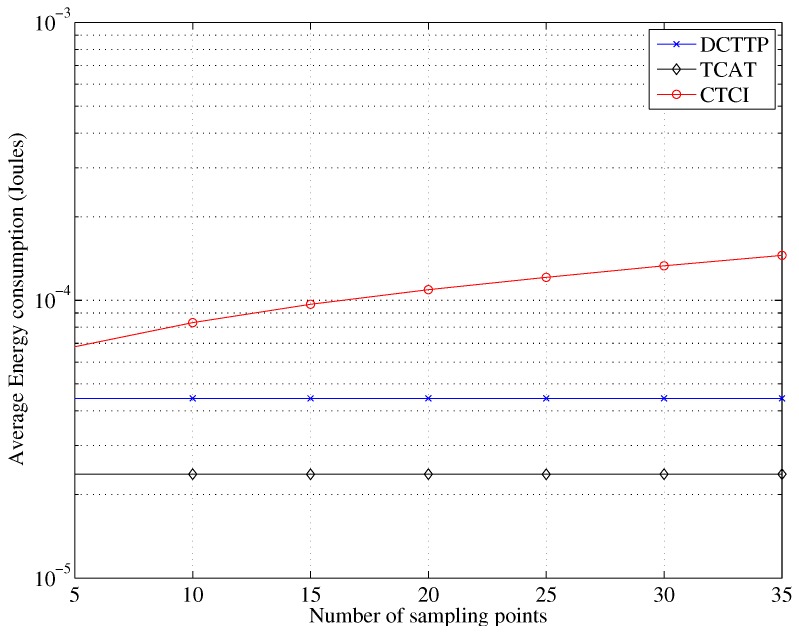
Impact of the sampling points on the average energy consumption.

Figure 20 depicts that the error distance of TCAT and CTCI decreases as the number of sample points increases. Observe that as the number of sample points is larger than 15, the error distance becomes stable. Figure 21 and Figure 22 depict that the data size and energy consumption of CTCI are directly influenced by the number of the sample points. Therefore, there is a trade-off between the error distance and the energy consumption on the number of the sample points in CTCI.

### 5.5. Noise

**Figure 23:**
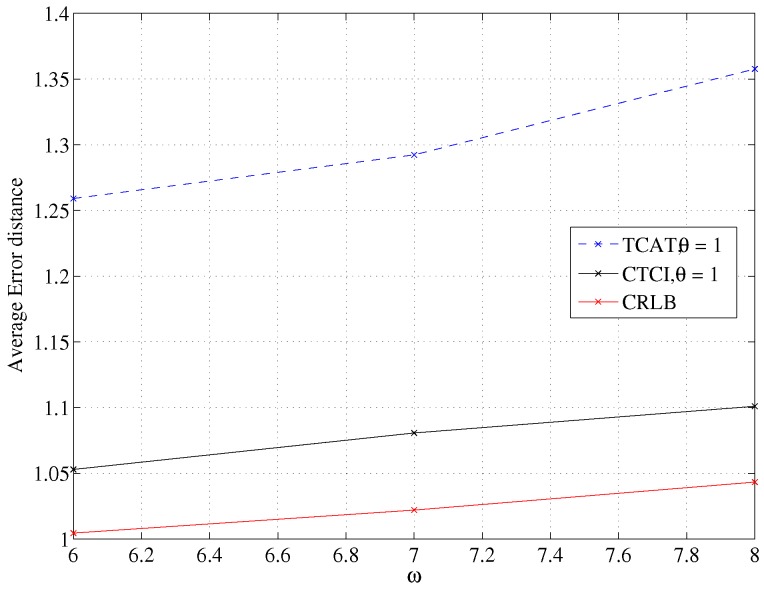
Impact of noise on the average error distance.

Because of the limited capability of a sensor and a extremely small sample size for particle filtering, the fundamental estimation problems are to estimate the distance between the reference sensors and the target and to determine the property of the signal (e.g., the signal arrival angle). In a nutshell, accurate tracking is highly related to precise TOA/AOA measurements and the signal processing capability. In this set of experiments, we explore the impact of measurement noise on system performance. With the variance of angle estimation $\sigma_{\theta }=1$ and with varying the uncertainty of distance estimation $\sigma_{d}$, as shown in Figure 23, the increment of the measurement noise leads to a larger error distance of TCAT and CTCI. Observe that with an increasing measurement uncertainty $\sigma_{d}$, the performance of the proposed CTCI method tends to be close to the CRLB.

### 5.6. Compressing Bound

**Figure 24:**
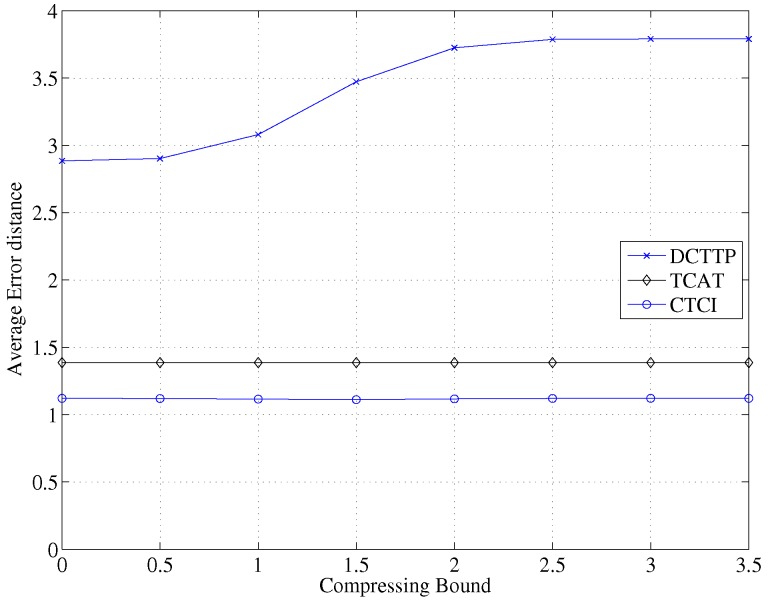
Impact of the compressing bound on the average error distance.

Figure 24 depicts that the error distance of DCTTP increases as the compressing bound increases. Because the sensed data of each active sensor node is compressed through the bitmap process, the distance between the active sensor node and the target may be represented by the average distance. Therefore, not only distortion may occur, but also the compressed data may influence the result of target tracking. The compressed data influences the weight of each active sensor node, so the reference value of the weight may decrease. Notice that in TCAT and CTCI, since the results of the positioning are not compressed through the bitmap process, the error distance is not influenced by the compressing bound. The bitmap process of the CTCI scheme is to obtain the information of measurement uncertainties in the original measurement data.

**Figure 25:**
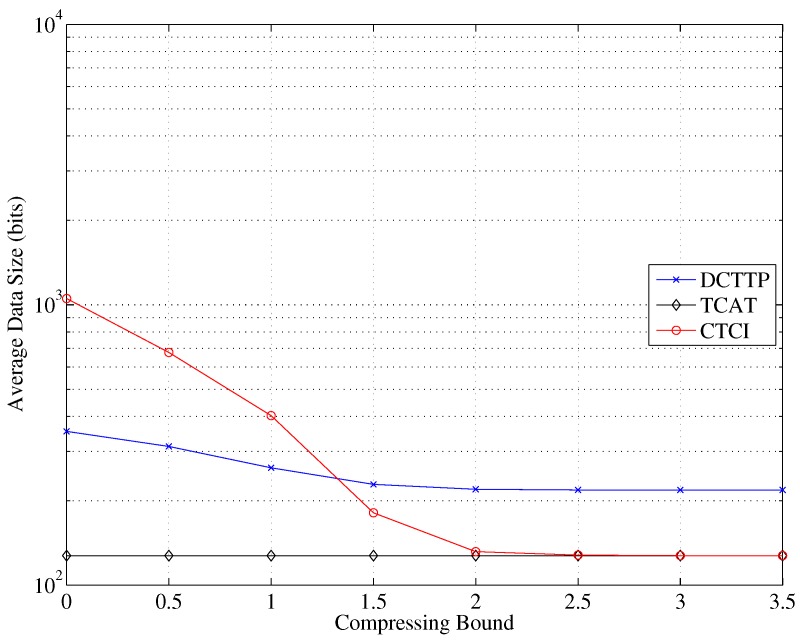
Impact of the compressing bound on the average data size.

**Figure 26:**
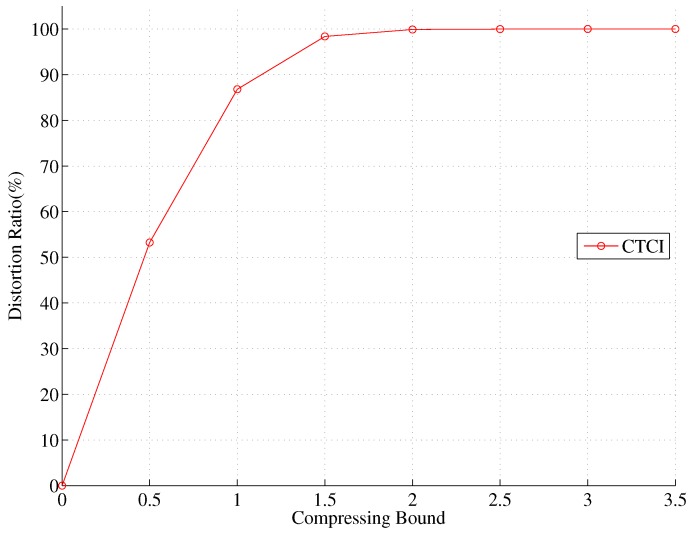
Impact of the compressing bound on the distortion ratio.

**Figure 27:**
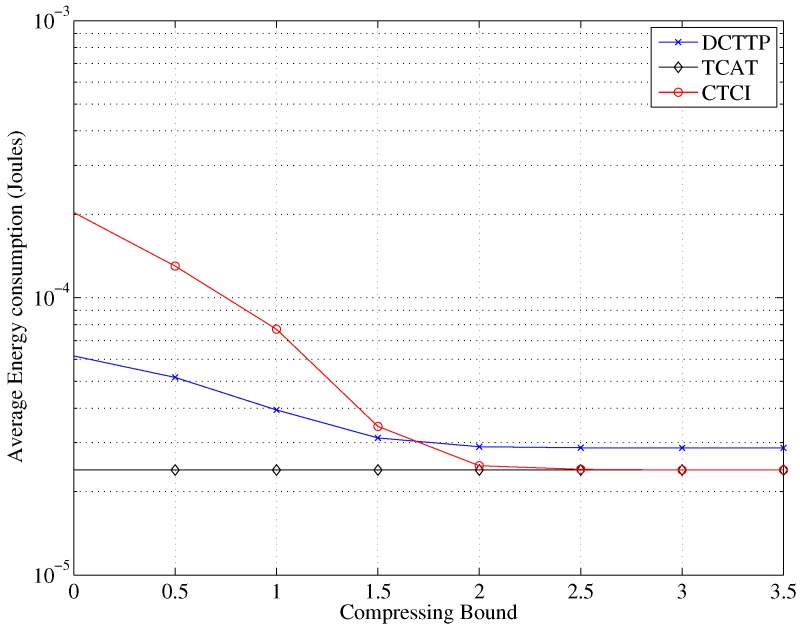
Impact of the compressing bound on the average energy consumption.

Observe that in Figure 25 when the compressing bound is zero, the data of DCTTP and CTCI will be reserved completely. As expected, the data size of DCTTP and CTCI decreases as the compressing bound increases. When the compressing bound is larger than two, the data are almost fully represented by the average value, and the error distance of DCTTP is saturated to a large estimation error. Define the distortion ratio as the portion of original sample points, which are replaced by the average value (*i.e.*, using the compressing bound to control the number of the remaining sample points). As shown in Figure 26, the distortion ratio of CTCI increases as the compressing bound increase. Figure 27 shows that if sensors with DCTTP and CTCI transmit their original data that are not compressed through the bitmap process, the cost of the energy consumption will become considerable. As depicted in Figure 25 and Figure 27, with a large compression bound, the TCAT scheme can be considered as a special case of CTCI, which compresses the samples to the mean.

## 6. Conclusions

This paper presents an information compression scheme for target tracking. Comparing the proposed CTCI method to the TCAT and DCTTP methods, the simulation results show that the proposed CTCI scheme can adaptively control the estimation performance and closely reflect the relationship between the distortion of tracking results and energy consumption. Considering the system parameters (e.g., transmission range, the number of sensor nodes, compressing bound, the number of sub-cluster size and the number of sampling points), at the slight cost in data size and energy consumption, the tracking accuracy of CTCI is superior to those of TCAT (improved by 20%) and DCTTP (improved by 100%). Therefore, many trade-offs between the system parameters need to be considered in each scheme. In future work, we will explore a more effective and robust compression scheme, evaluate the compression complexity and quality and adjust the tactics of the two-level clustering to further reduce the data size, maintain the desired estimation quality and achieve energy conservation.

## Figures and Tables

**Table 1:** An example for the fixed point representation.

Value									−5.678								
Code	1	1	0	0	1	0	1		1	0	1	0	1	1	0	1	1
Meaning	±	$2^{2}$	$2^{1}$	$2^{0}$	$2^{2}$	$2^{1}$	$2^{0}$	.	$2^{-1}$	$2^{-2}$	$2^{-3}$	$2^{-4}$	$2^{-5}$	$2^{-6}$	$2^{-7}$	$2^{-8}$	$2^{-9}$
Bit list	1	2	3	4	5	6	7		8	9	10	11	12	13	14	15	16

**Table 2:** The values of the simulation parameters.

Parameter	Value
The sensing field	100 × 100 $m^{2}$
Base station location	(50,50)
Number of sensors	200
Transmission range	15 m
Number of samples	15
Sub-cluster size *n*	2
TOA measurement deviation $\sigma_{w}$	2
AOA measurement deviation $\sigma_{\theta }$	2
Compressing bound parameters	0.8
